# Comprehensive Expression Profile Analysis of Neutrophil Extracellular Trap-Affected Genes in Gastric Cancer Cells and the Clinical Significance of lncRNA NEAT1-Related Signaling

**DOI:** 10.3389/fonc.2022.798531

**Published:** 2022-05-19

**Authors:** Changjian Li, Xiaoming Zou, Qingxin Cai, Jiacheng Li, Shifeng Yang, Ange Zhang, Chongyan Chen, Lei Zhu

**Affiliations:** ^1^ Department of Gastrointestinal Surgery, The Second Affiliated Hospital of Harbin Medical University, Harbin, China; ^2^ Department of Pharmacy, The First Specialized Hospital of Harbin, Harbin, China

**Keywords:** neutrophil extracellular traps, gastric cancer, RNA sequencing, *NEAT1*, *RAB3B*

## Abstract

**Background:**

Gastric cancer (GC) is the fifth most common malignant tumor and the third leading cause of cancer-related deaths worldwide. Neutrophil extracellular traps (NETs) can enhance the invasion of GC cells and are associated with poor prognosis in patients. However, its mechanism of action is not completely understood.

**Methods:**

The content of NETs in the peripheral blood of patients with GC was detected by enzyme-linked immunosorbent assay. GC AGS cells were treated with or without NETs for 24 h. High-throughput RNA sequencing was performed to screen differentially expressed long non-coding RNAs (lncRNAs), microRNAs (miRNAs), and messenger RNAs (mRNAs). Real-time polymerase chain reaction (PCR) was used to verify gene expression. A competing endogenous RNA (ceRNA) regulatory network was constructed. Modules were screened using the molecular complex detection (MCODE) plug-in. Gene Ontology and Kyoto Encyclopedia of Genes and Genomes enrichment analyses were performed using the genes in the network. The role and clinical significance of the lncRNA *NEAT1*-related signaling pathway were validated.

**Results:**

The content of NETs in the patients with GC was significantly higher than that in healthy controls and was also higher in patients with high-grade (stages III and IV) GC. NETs promoted the invasion of AGS cells. A total of 1,340 lncRNAs, 315 miRNAs, and 1,083 mRNAs were differentially expressed after NET treatment. The expression of five genes was validated using real-time PCR, which were in accordance with the RNA sequencing results. A ceRNA regulatory network was constructed with 1,239 lncRNAs, 310 miRNAs, and 1,009 mRNAs. Four genes (*RAB3B*, *EPB41L4B*, *ABCB11*, and *CCDC88A*) in the ceRNA network were associated with patient prognosis, with *RAB3B* being the most prominent and with signaling among the lncRNA *NEAT1*, the miRNA miR-3158-5p, and *RAB3B*. *NEAT1* was upregulated in AGS cells after NET treatment. RNA interference of *NEAT1* inhibited the invasion of AGS cells induced by NETs, inhibited miR-3158-5p expression, and promoted *RAB3B* expression. *NEAT1* and *RAB3B* expression were positively correlated in patients with GC. Furthermore, *RAB3B* was upregulated and miR-3158-5p was downregulated in GC tissues compared with adjacent normal tissues, which was also associated with cancer stage.

**Conclusion:**

This study provides a comprehensive analysis of differentially expressed genes in NET-treated GC cells and validated the clinical significance of *NEAT1*-related signaling.

## Background

Gastric cancer (GC) is one of the most common and aggressive cancers worldwide. Currently, although the clinical diagnosis and treatment of GC have gradually advanced, the 5-year survival rate is still low (1). Therefore, it is particularly important to explore the pathogenesis and progression mechanisms of GC.

Inflammation is a hallmark of cancer (2, 3). Immune cells and factors released by immune cells are essential for cancer (4–6). Neutrophil extracellular traps (NETs) are DNA networks that are released by neutrophils. Many studies have shown that tumor cells can recruit neutrophils and stimulate them to generate NETs (7, 8). The levels of NETs in patients with advanced breast cancer are significantly higher than those in patients with early-stage cancer (9). In Ewing’s sarcoma, NETs exist only in patients with metastasis (10). Zhang et al. explored the diagnostic and prognostic value of NETs in patients with GC. They found that high peripheral blood NET levels were associated with lymph node metastasis in patients with GC. Moreover, peripheral blood NET levels were negatively correlated with short-term efficacy and were an independent prognostic marker for patients with GC. Higher baseline peripheral blood NET levels correlated with worse progression-free survival (PFS) (11). These studies proved that high levels of NETs are associated with cancer progression, invasion, and poor prognosis. However, the mechanisms of NET formation in GC have not been fully elucidated.

There are many types of RNAs in the human body, which can be divided into coding and non-coding RNA (ncRNA), depending on whether they encode proteins. A great quantity of studies have revealed the important role of ncRNAs in GC, which mainly include microRNAs (miRNAs) and long ncRNAs (lncRNAs). For example, overexpression of miR-101 promoted GC progression both *in vivo* and *in vitro* (12). miR-96-5p was highly expressed in GC cells and the plasma of patients with GC. Moreover, miR-96-5p promotes GC cell proliferation by directly inhibiting *FOXO3* expression (13). lncRNAs are differentially expressed in GC samples (14, 15). The lncRNA–miRNA co-expression relationship was observed in the plasma of patients with GC (16). Qi et al. constructed an lncRNA-based competing endogenous RNA (ceRNA) network in GC and identified potential therapeutic and prognostic markers for GC (17). Some lncRNAs have been shown to exert their functions by targeting miRNAs. For example, enhanced expression of the lncRNA *NORAD* is correlated with poor prognosis in patients with GC. A mechanistic study found that overexpression of *NORAD* could promote the growth of GC cells by regulating the miR-608/*FOXO6* pathway (18). The lncRNA *LINC00629* is downregulated in GC tissues and cells. Overexpression of *LINC00629* inhibited GC progression by regulating the miR-196b-5p/*AQP4* signaling pathway (19). These findings indicate that lncRNA- and miRNA-derived signaling pathways are important in GC.

The effect of NETs on ncRNAs in GC is not well understood. In this study, we comprehensively analyzed the expression profile of NET-affected genes (lncRNA, miRNA, and mRNA) in GC cells and verified the clinical significance of lncRNA *NEAT1*-related signaling.

## Materials and Methods

### Patients

Health control and GC patients were recruited at the Second Affiliated Hospital of Harbin Medical University of China between 2019 and 2021. All GC patients were diagnosed by gastroscopy pathology. Tumor–node–metastasis (TNM) staging was based on postoperative pathology and the 8th AJCC Guidelines. The exclusion criteria include age <18 years, among others (20). The present study was approved by the Research Ethics Committee.

### Cell Line and Cell Culture

The GC line of human (AGS) was purchased from the Chinese Academy of Sciences.

Cells were cultured in 10% fetal bovine serum (FBS, #04-001-1ACS, BI) with penicillin (100 U/ml) and cultured in a humid environment at 37°C under 5% CO_2_ and 95% air.

### Isolation of Neutrophils and Extraction of NETs

Cell-free NETs were isolated from neutrophils of patients with GC. Briefly, neutrophils (1×10^7^/ml) were treated with 500 nM PMA in a cell culture dish for 4 h at 37°C in a 5% CO_2_ humidified chamber. Ice-cold 1×PBS (10 ml) was added to wash down the cell layer of neutrophils at the bottom of the culture dish to obtain the NET formation after the supernatant was abandoned. The NETs were stored at −80°C and used for further experiments. NETs stimulated GC cells by adding 0.5 µg/ml NETs to GC AGS cells in a Petri dish and acting in an incubator for 24 h (21).

### Cell Invasion Assay

Cell invasion assay was verified by Transwell. The sequence of siNEAT1 and siNC is as follows: siNEAT1: sense 5’-GACCGUGGUUUGUUACUAUdTdT-3’, antisense 5’-AUAGUAACAAACCACGGUCdTdT-3’; siNC: sense 5’-UUCUCCGAACGUGUCACGUT-3’, antisense 5’-ACGUGACACGUUCGGAGAAT-3’. Matrigel (40 µl; BD Biosciences, San Jose, CA, USA) was added to the precooled Transwell chamber and incubated at 37°C for 2 h to solidify. The AGS cells were transfected with siNC or siNEAT1, with or without NETs, and 100,000 cells were counted and placed in the upper chamber with an aperture of 8 µM pore size (BD Biosciences, San Jose, CA, USA). Cell culture medium containing 10% FBS was added to the lower chamber. After incubation for 24 h at 37°C, it was fixed and stained with crystal violet staining solution and counted under the microscope.

### ELISA

We measured plasma MPO–DNA complex by using capture ELISA. Briefly, 96-well microtiter plates were coated with anti-MPO monoclonal antibody (1:1,000, ab25989, Cambridge, UK). After blocking in 1% BSA, patient plasma was combined with Quant-iT PicoGreen dsDNA (Invitrogen, USA). After the peroxidase substrate (Roche, China) was added and incubated at 37°C for 40 min, the optical density was measured at 405 nm.

### Real-Time PCR Analysis

AGS cells and tissues were extracted using the total RNA using TRIzol reagent (Invitrogen, CA, USA). Transcriptor the first strand cDNA synthesis was used transcriptase kit (Roche, Mannheim, Germany) and reverse transcription was performed using HiScript R II Q RT SuperMixfor qPCR (Vazyme Biotech Co., Ltd., Nanjing, China). The primers are shown in **Table 1**. Relative expression was analyzed using the 2^−ΔΔCT^ method.

**Table 1:** The primers for real-time PCR.

Gene name	Forward (5’-3’)		Reverse (5’-3’)
NEAT1 miR-3158-5p	CCAAGACAGCCTGTTTCAGA ACACTCCAGCTGGGCCTGC		GATGCTGATCTGCTGCGTAT CTCAACTGGTGTCGTGGA
RAB3B LINC00659 LINC01996 hsa-miR-1246 INHBA HOXC5 β-actin	CGGGTGAAACTGCAGATCT AGCGCTGACCTGTGTAAAGA GAACCTGCCGTTGTTTGTCA ACACTCCAGCTGGGAATGG TTGCTCCCTCTGGCTATCAT GATGTACAGTCAGAAGGCGG TGGATCAGCAAGCAGGAGTA		CTGAGTAGCCCAGTCTTGGA GGGAGCCTCCTGTTAAGCATC CAGGGTGGAATCTGAGTGT CTCAACTGGTGTCGTGGA ACGATTTGAGGTTGGCAAAG GTTTGGTCATCCACGGGTAA TCGGCCACATTGTGAACTTT

### High-Throughput RNA Sequencing and Module Analysis

RNA sequencing and analysis were conducted by Genedenovo Bioinformatics Technology Co., Ltd (Guangzhou, China). We identified mRNA and lncRNA with a fold change ≥2 and an FDR < 0.05, in comparison with significant DEGs, and miRNA with a fold change ≥2 and *p* < 0.05 (22).

### Construction of the lncRNA–miRNA–mRNA Network and Functional Enrichment Analysis

The ceRNA network was constructed by ceRNA theory. RNAhybrid (V2.1.2) + svm_light (v6.01), Miranda (v3.3a), and TargetScan (version 7.0) were used for target gene prediction of miRNA. Then, the resulting intersection genes were selected as the target genes of miRNA. Expression correlation among lncRNA–mRNA–miRNA was evaluated using the Spearman Rank correlation coefficient (SCC < −0.7) and the Pearson correlation coefficient (PCC > 0.9). As a result, only the gene pairs with a *p* < 0.05 were selected. The lncRNA–miRNA–mRNA network and functional enrichment analyses were visualized using Cytoscape software (v3.6.0) (23).

### Luciferase Assay

mRNA miR-3158-5p was transfected with the constructed plasmid. Luciferase activity was analyzed using a dual-luciferase assay kit (Promega, Madison, USA) on a GloMax^®^ Discover System (Promega, Madison, WI, USA).

### Statistical Analysis

All data are presented as the mean ± SD. Statistical comparisons between two or among four groups were performed using *t*-test and one-way ANOVA, respectively. Chart generation was performed by GraphPad Prism 8.0 software. *p* < 0.05 was considered statistically significant.

## Results

### NETs Were Upregulated in the Peripheral Blood of GC Patients and Could Promote Invasion of GC Cells

**Figure 1:**
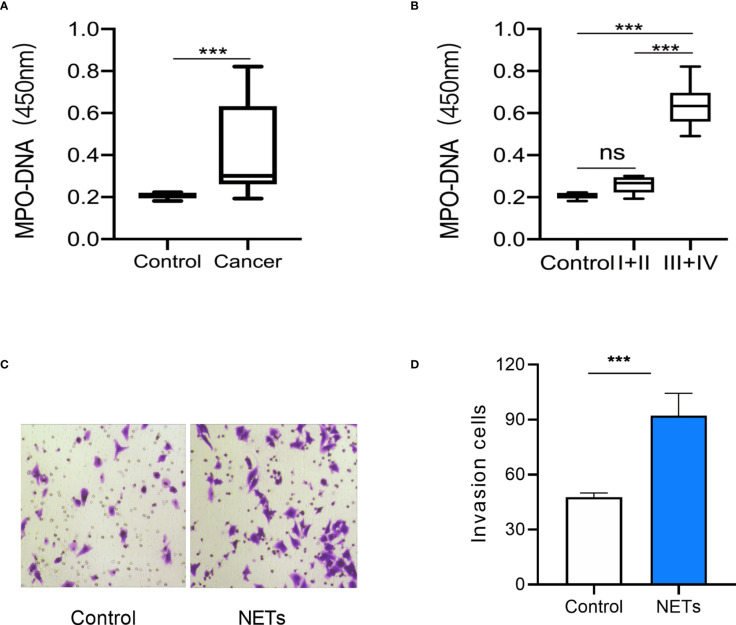
NETs expression in peripheral blood of gastric cancer patients and its effect on gastric cancer cells. **(A)** NETs expression in peripheral blood of GC patients and healthy controls. **(B)** NETs expression in peripheral blood of GC patients in different stages. **(C)** Representative images of the invasion assay. **(D)** The analysis results of invasive AGS cells treated by NETs. For panels **(A, B)**, Control group, *n* = 10; GC group, *n* = 41; I+II group, *n* = 20; III + IV group, *n* = 21; for panel **(D)**, *n* = 4; ***p < 0.001.

The levels of myeloperoxidase–DNA complexes in the peripheral blood of 41 patients with GC (10 with stage I, 10 with stage II, 19 with stage III, and 2 with stage IV) and 10 healthy controls were detected. NET expression was higher in patients with GC than in healthy controls (**Figure 1A**). We then observed an association between NET expression and GC stage. The results showed that NET expression was similar in stage I and II patients but higher in stage III and IV patients (**Figure 1B**). We then observed the effect of NETs on the invasion of GC cells. The results showed that NETs promoted the invasion of AGS cells (**Figures 1C, D**). It is suggested that the level of NETs in patients with advanced GC is higher.

### Gene Expression Profile in GC Cells Stimulated by NETs

**Figure 2:**
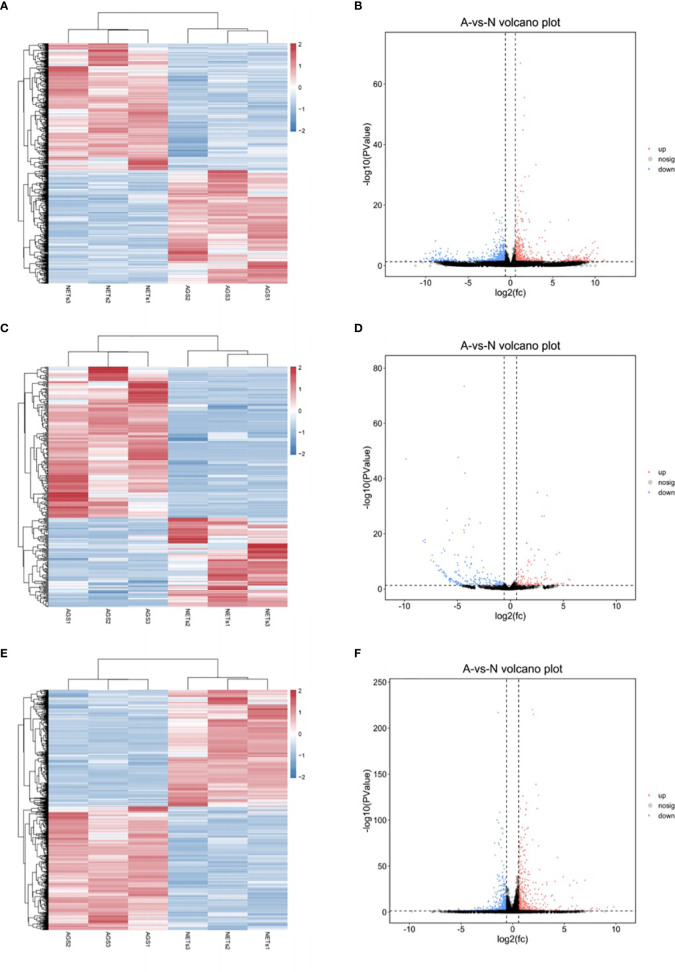
The differentially expressed genes in gastric cancer cells treated by NETs. **(A)** Heatmap of differentially expressed lncRNAs. **(B)** Volcano plots of differentially expressed lncRNAs. **(C)** Heatmap of differentially expressed miRNAs. **(D)** Volcano plots of differentially expressed miRNAs. **(E)** Heatmap of differentially expressed mRNAs. **(F)** Volcano plots of differentially expressed mRNAs. *p* < 0.05, |log2(fold change)| > 1.

Using high-throughput RNA sequencing, we detected the expression profiles of lncRNAs, miRNAs, and mRNAs in GC AGS cells stimulated by NETs. Heatmaps and volcano plots of expressed genes are shown in **Figure 2**. Differentially expressed RNAs detected between the control and NET-stimulated cells were determined with *p* < 0.05 and |log_2_(fold change)| > 1. There were 1,340 differentially expressed lncRNAs (708 upregulated after NET stimulation and 632 downregulated after NET stimulation, **Figures 2A, B** and **Supplementary Data 1**), 315 differentially expressed miRNAs (117 upregulated and 198 downregulated, **Figures 2C, D** and **Supplementary Dataset 2**), and 1,083 differentially expressed mRNAs (526 upregulated and 557 downregulated, **Figures 2E, F** and **Supplementary Dataset 3**).

### Verification of Gene Expression by Real-Time PCR

**Figure 3:**
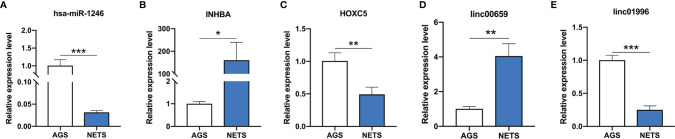
The expression of genes validated by real-time PCR. **(A)** The expression of hsa-miR-1246 in NET-treated GC cells. **(B)** The expression of INHBA in NET-treated GC cells. **(C)** The expression of HOXC5 in NET-treated GC cells. **(D)** The expression of linc00659 in NET-treated GC cells. **(E)** The expression of linc01996 in NET-treated GC cells. *n* = 3, *p < 0.05, **p < 0.01, ***p < 0.001.

The lncRNA *LINC00659* (ENST00000667589) and the mRNA *INHBA* were upregulated and the lncRNA *LINC01996* (ENST00000573270), the miRNA hsa-miR-1246, and the mRNA *HOXC5* were downregulated in GC cells stimulated by NETs (**Figures 3A** **–E**). These results are consistent with the RNA sequencing results.

### Construction and Enrichment Analysis of the ceRNA Network

**Figure 4:**
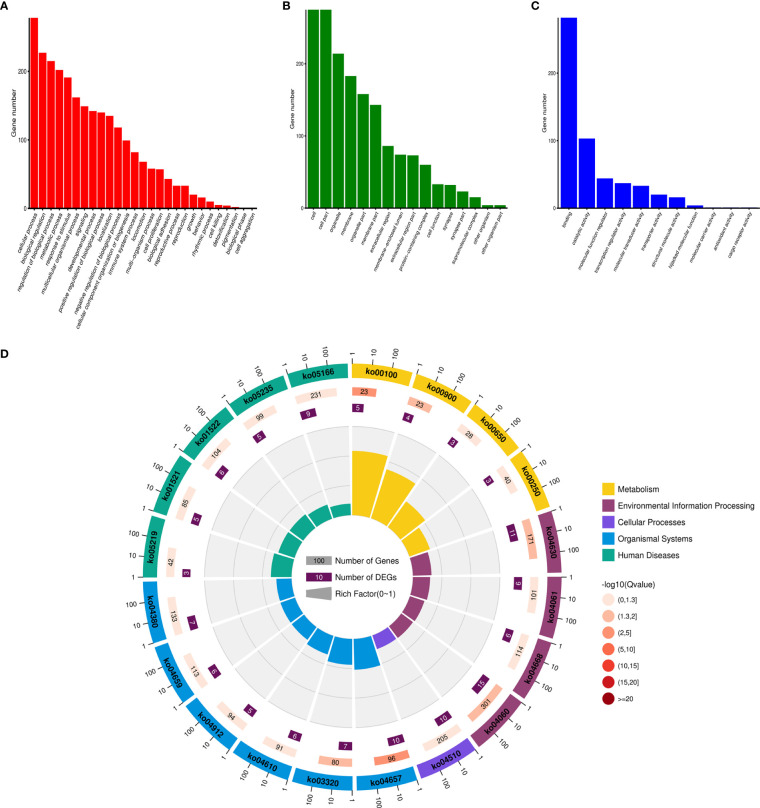
The enrichment analysis of the ceRNA network. **(A)** Biological Process GO enrichment analysis of the ceRNA network. **(B)** Cellular Component GO enrichment analysis of the ceRNA network. **(C)** Molecular Function GO enrichment analysis of the ceRNA network. **(D)** KEGG enrichment analysis of the ceRNA network.

Based on the expression of genes and miRNA target prediction results, an lncRNA–miRNA–mRNA ceRNA network was constructed. The ceRNA network contained 7,520 edges and 789 nodes, including 1,239 lncRNAs, 310 miRNAs, and 1,009 mRNAs (**Supplementary Datasets 4, 5**). Enrichment analysis of the mRNAs involved in the ceRNA network was performed and was enriched in pathways including the IL-17 signaling pathway (**Figure 4**).

### Module Analysis of the ceRNA Network

**Figure 5:**
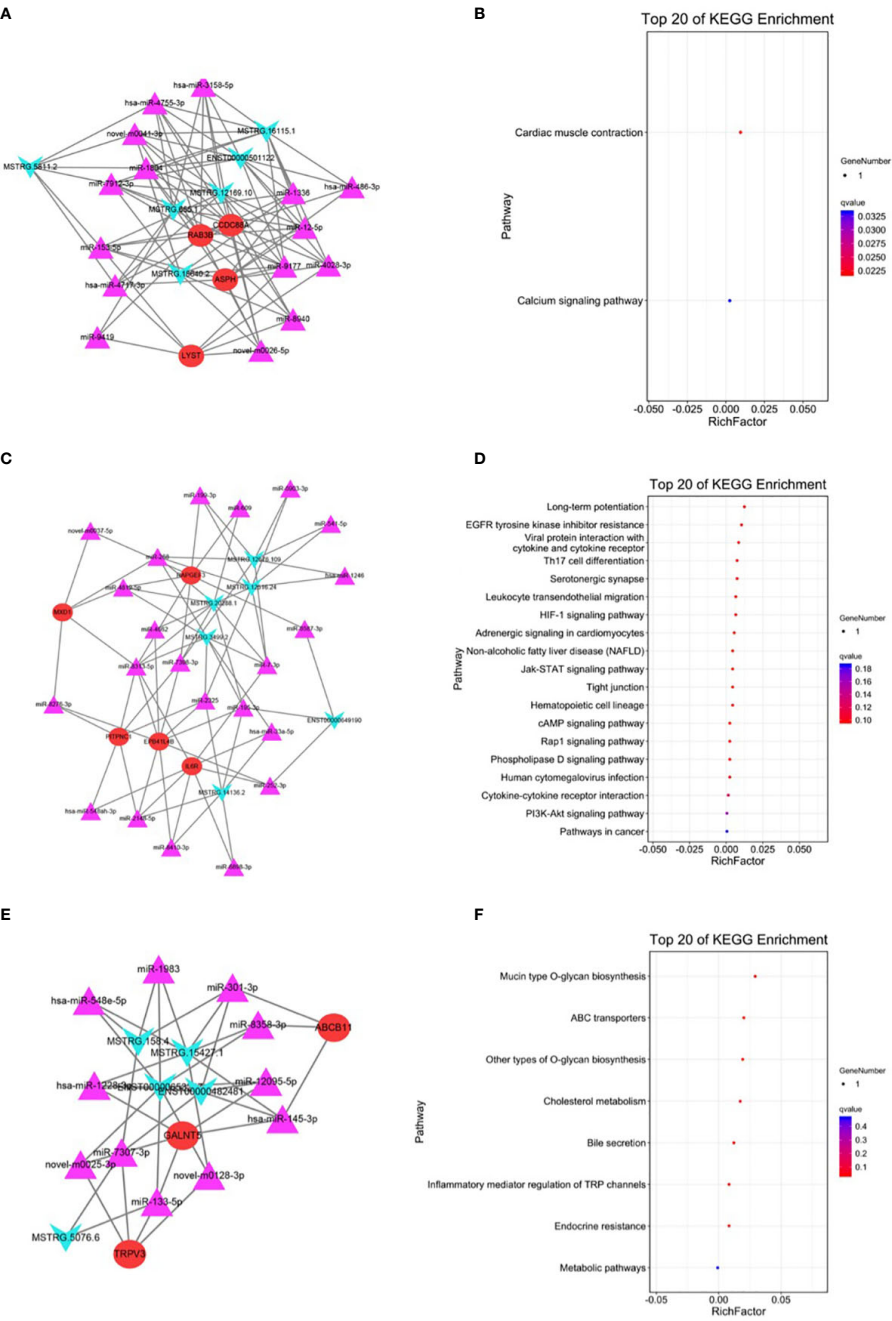
Module analysis and enrichment analysis of the ceRNA network. **(A)** The ceRNA network of module 1. **(B)** KEGG enrichment analysis of module 1. **(C)** The ceRNA network of module 2. **(D)** KEGG enrichment analysis of module 2. **(E)** The ceRNA network of module 3. **(F)** KEGG enrichment analysis of module 3. For panels **(A, C, E)**, arrow nodes represent lncRNAs, triangular nodes represent miRNAs, and square nodes represent mRNAs.

The top three modules generated using MCODE are presented in **Figure 5**. Module 1 contained 25 nodes and 94 edges and was associated with the calcium signaling pathway (**Figures 5A, B**). Module 2 contained 33 nodes and 73 edges and was associated with the cAMP signaling pathway (**Figures 5C, D**). Module 3 contained 19 nodes and 38 edges and was associated with inflammatory mediators of transient receptor potential (TRP) channels (**Figures 5E, F**).

### Association Between mRNAs in the ceRNA Network and Overall Survival of Patients With GC

**Figure 6:**
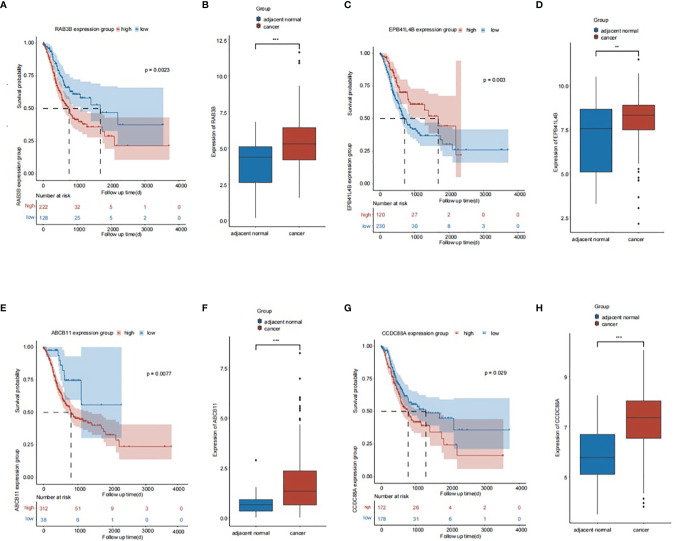
The association of genes in the ceRNA network and prognosis of gastric cancer (GC) patients. **(A)** The association of RAB3B and prognosis of GC patients. **(B)** The expression of RAB3B in GC tissues. **(C)** The association of EPB41L4B and prognosis of GC patients. **(D)** The expression of EPB41L4B in GC tissues. **(E)** The association of ABCB11 and prognosis of GC patients. **(F)** The expression of ABCB11 in GC tissues. **(G)** The association of CCDC88A and prognosis of GC patients. **(H)** The expression of CCDC88A in GC tissues. **p < 0.01, ***p < 0.001.

**Figure 7:**
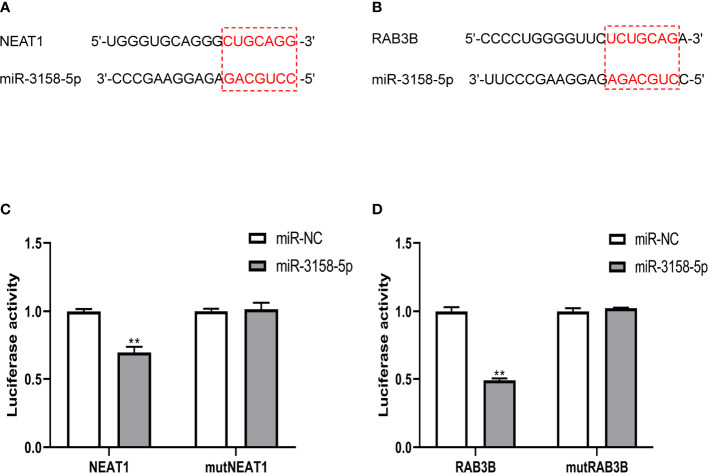
NEAT1 and RAB3B were direct targets of miR-3158-5p. **(A)** The binding site sequence between *NEAT1* and miR-3158-5p. **(B)** The binding site sequence between RAB3B and miR-3158-5p. **(C)** Luciferase reporter assay verified the direct binding effect between *NEAT1* and miR-3158-5p. **(D)** Luciferase reporter assay verified the direct binding effect between *RAB3B* and miR-3158-5p. For panels C and D, **p < 0.01 vs. miR-NC.

The overall survival (OS) rate of GC was predicted using mRNAs in the ceRNA network and bioinformatic methods. In the ceRNA network, four genes were associated with the OS of patients with GC, among which *RAB3B* was the most prominent. *RAB3B*, *ABCB11*, and *CCDC88A* expression levels were negatively associated with OS, whereas *EPB41L4B* expression was positively associated with OS (**Figure 6**). In addition, signaling between the lncRNA *NEAT1*, the miRNA miR-3158-5p, and the mRNA *RAB3B* was noted in the ceRNA network. Luciferase assay revealed the direct interaction between miR-3158-5p and *NEAT1/RAB3B* and also showed that miR-3158-5p could directly bind to *NEAT1* and *RAB3B* (**Figure 7**).

### NETs Promote the Invasion of GC Cells by Upregulating NEAT1

**Figure 8:**
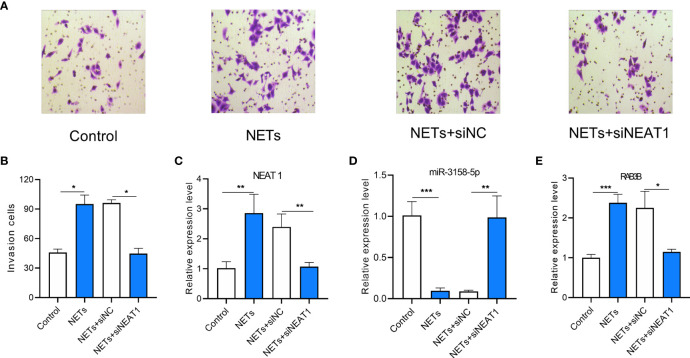
The effect of lncRNA NEAT1 knockdown on invasion of gastric cancer (GC) and miR-3158-5p/RAB3B expression in gastric cancer cells stimulated by NETs. **(A)** Representative images of Transwell invasion assay. **(B)** The analysis results of invasive AGS cells treated by NETs and knockdown of lncRNA NEAT1. **(C)** The expression of lncRNA NEAT1 in AGS cells. **(D)** The expression of miR-3158-5p in AGS cells. **(E)** The expression of RAB3B in AGS cells. For Panels **(A, B)** *n* = 8, *p < 0.05, for panels C–E, *n* = 3, *p < 0.05, **p < 0.01, ***p < 0.001.

The invasion ability of GC cells treated with NETs was measured using a Transwell invasion assay. *NEAT1* knockdown inhibited the invasion ability of NET-treated GC cells (**Figures 8A, B**). The expression levels of *NEAT1*, miR-3158-5p, and *RAB3B* were assessed using real-time PCR. NETs promoted *NEAT1* and *RAB3B* expression and inhibited miR-3158-5p expression. *NEAT1* knockdown inhibited *NEAT1* expression and attenuated the effect of NETs on miR-3158-5p and *RAB3B* expression (**Figures 8C** **–E**). Therefore, NETs promote the invasion of GC cells by the *NEAT1/*miR-3158-5p*/RAB3B* axis.

### Clinical Associations Between NETs and the NEAT1/miR-3158-5p/RAB3B Axis in Human GC Samples

**Figure 9:**
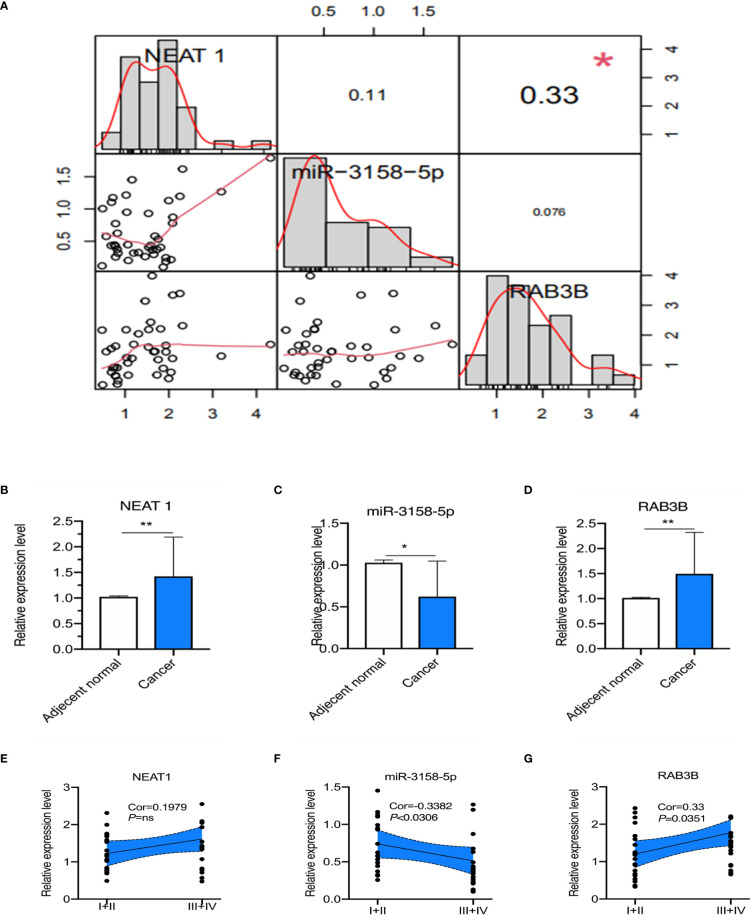
The correlation and prognosis effect among lncRNA NEAT1, miR-3158-5p, and RAB3B expression in gastric cancer (GC) patients. **(A)** The correlation among lncRNA NEAT1, miR-3158-5p, and RAB3B. **(B)** The expression of lncRNA NEAT1 in GC and adjacent normal tissues. **(C)** The expression of miR-3158-5p in GC and adjacent normal tissues. **(D)** The expression of RAB3B in GC and adjacent normal tissues. **(E)** The expression of lncRNA NEAT1 in GC patients of different stages. **(F)** The expression of miR-3158-5p in GC patients of different stages. **(G)** The expression of RAB3B in GC patients of different stages. For Panels **(A–D)**, *n* = 41, *p < 0.05, **p < 0.01; for panels **(E–G)**, *n* = 20–21.

Finally, we observed the clinical significance of the *NEAT1*/miR-3158-5p/*RAB3B* axis. *NEAT1* expression was positively correlated with *RAB3B* in patients with GC (**Figure 9A**). miR-3158-5p expression was negatively associated with *NEAT1* and *RAB3B*, but without statistical significance (**Figure 9A**). *NEAT1* and *RAB3B* expression were higher and miR-3158-5p was lower in GC tissues than adjacent normal tissues (**Figures 9B** **–D**). miR-3158-5p was negatively associated and *RAB3B* was positively associated with cancer stage, while *NEAT1* was positively associated with cancer stage, but without statistical significance (**Figures 9E** **–G**).

## Discussion

In the present study, we validated the effect of NETs on the invasion ability of GC cells. It was in accordance with current findings that NETs could promote the invasion of cancer cells (24–26). However, little is known about the related mechanisms, especially the involvement of ncRNAs. We then screened the differentially expressed genes in NET-stimulated GC cells. A total of 2,558 genes (1,340 lncRNAs, 315 miRNAs, and 1,083 mRNAs) were differentially expressed in GC cells after NET treatment (**Figures 1**, **2**). Five of these genes were validated using real-time PCR (**Figure 3**), and results were in accordance with the RNA sequencing results.

Based on the gene expression analysis and miRNA target prediction, we constructed a ceRNA network. The mRNAs involved in the network were enriched in pathways including the IL-17 signaling pathway, steroid biosynthesis, and terpenoid backbone biosynthesis (**Figure 4**). The relationship between some of these pathways and NETs has been proven. For example, serum IL-17 levels were higher in patients with GC than in healthy controls, which is a potential diagnostic biomarker for GC (27). The relationship between IL-17 and NETs has been investigated in pancreatic cancer. IL-17 can induce NET formation, which mediates resistance to pancreatic cancer (28). We then performed module analysis of the ceRNA network. KEGG and GO enrichment analysis was conducted on the top three modules (**Figure 5**). The mRNA-enriched pathways included the cAMP signaling pathway, the HIF-1 signaling pathway, and inflammatory mediators of TRP channels (29–32).

To evaluate the clinical significance of the ceRNA network, the prognostic value of mRNAs in the ceRNA network was analyzed. *RAB3B*, *EPB41L4B*, *ABCB11*, and *CCDC88A* were correlated with the prognosis of patients with GC. *RAB3B* was the most prominent among the four genes (**Figure 6**). *RAB3B* expression was higher in glioma tissues, which correlated with the grade of glioma. *RAB3B* knockdown inhibits proliferation and promotes apoptosis of glioma cells (33). *RAB3B* expression is elevated in patients with prostate cancer and is an important regulator of cancer progression (34). Therefore, *RAB3B* will likely act as an oncogene in GC, which may be regulated by a ceRNA mechanism.

In the ceRNA network, *NEAT1* regulated *RAB3B* by sponging miR-3158-5p. *NEAT1* has been reported to promote GC progression (35). We measured the expression of *NEAT1* on the invasion of GC cells stimulated by NETs. NETs can enhance the invasion of GC cells and upregulate *NEAT1* and *RAB3B* expression, whereas they downregulate miR-3158-5p expression (**Figure 8**). The influence of NETs on GC cells could be attenuated by knocking down *NEAT1* (**Figure 8**).

NETs may be used as a biological marker in the diagnosis and prediction of GC prognosis (36). In addition, RNAs have become the direct mechanisms for healthy cells to transform into tumor cells and play vital roles in cancer diagnosis and prognosis (37, 38). Therefore, we further evaluated the expression and association of these three genes in the tissues of patients with GC.

*NEAT1* was positively correlated with *RAB3B* in patients with GC, while miR-3158-5p was negatively associated with *NEAT1* and *RAB3B*, without statistical significance (**Figure 9A**). Moreover, the expression levels of *NEAT1* and *RAB3B* were higher and miR-3158-5p was lower in GC than in adjacent normal tissues (**Figures 9B** **–D**). miR-3158-5p was negatively associated and *RAB3B* was positively associated with cancer stage, while *NEAT1* was positively associated with cancer stage, but without statistical significance. The expression levels of *NEAT1* (without statistical significance) and *RAB3B* were higher and that of miR-3158-5p was lower in stage III/IV than in stage I/II patients (**Figures 9E** **–G**). The reason for the lack of statistical significance may be the limited sample size. These findings indicate that ceRNA triplets are involved in the progression of GC. More basic and clinical in-depth experiments are needed to reveal the regulation of NETs in the ceRNA network in GC.

In conclusion, this study provides a comprehensive analysis of differentially expressed genes in NET-treated GC cells and validated the clinical significance of *NEAT1*-related signaling.

## Data Availability Statement

The datasets presented in this study can be found in online repositories. The names of the repository/repositories and accession number(s) can be found at: NCBI GEO, accession no: GSE188741.

## Ethics Statement

The studies involving human participants were reviewed and approved by the Research Ethics Committee of Second Affiliated Hospital of Harbin Medical University. The patients/participants provided their written informed consent to participate in this study.

## Author Contributions

CL and XZ designed the study. CL, JL and SY performed the experiment. AZ, LZ, and CC analyzed the data. CL and QC wrote the manuscript. All authors contributed to the article and approved the submitted version.

## Funding

This project was supported by the National Natural Science Foundation of China (81672355).

## Conflict of Interest

The authors declare that the research was conducted in the absence of any commercial or financial relationships that could be construed as a potential conflict of interest.

## Publisher’s Note

All claims expressed in this article are solely those of the authors and do not necessarily represent those of their affiliated organizations, or those of the publisher, the editors and the reviewers. Any product that may be evaluated in this article, or claim that may be made by its manufacturer, is not guaranteed or endorsed by the publisher.
